# Mesenchymal Stem Cells Accelerate the Remodeling of Bladder VX2 Tumor Interstitial Microenvironment by TGFβ1-Smad Pathway

**DOI:** 10.7150/jca.30788

**Published:** 2019-07-25

**Authors:** Qingya Yang, Jun Chen, Yaofeng Zhu, Zhishun Xu

**Affiliations:** 1 Department of Urology, Qilu Hospital (Qingdao), Shandong University, Qingdao 266035, China.; 2 Department of Urology, Qilu Hospital, Shandong University, Jinan 250012, China

**Keywords:** Mesenchymal stem cells, tumor, stroma remodeling

## Abstract

**Background:** Mesenchymal stem cells (MSCs) have been proved to be able to differentiate into cells that are conducive to tumor growth and invasion. The mechanism is not clear. This present study was aimed to find out whether TGFβ1-Smad pathway was involved in this process.

**Methods:** For the* in vitro* experiment, five groups of MSCs were cultured to test whether VX2 culture supernatant could induce the differentiation of MSCs into myofibroblasts. And then transforming growth factor β1(TGFβ1) receptor or Smad2 of MSCs were blocked by RNA interference technique to test whether TGFβ1-Smad pathway was involved in the differentiation. In the animal experiment, different kinds of MSCs were co-inoculated with VX2 cells in bladder to test whether the blockage of TGFβ1 receptor or Smad2 of MSCs could affect the expression of TGFβ1, epidermal growth factor (EGF), fibroblast activation protein alpha (FAPa), and matrix metalloprotein 9 (MMP9) in five animal groups.

**Results:** VX2 culture supernatant could up-regulate the expression of α-SMA and Vimentin in MSCs, which indicated that VX2 culture supernatant could induce the differentiation of MSCs into myofibroblasts. Either the Blockage of TGFβ1 receptor or Smad2 of MSCs could lead to decreased expression of α-SMA and Vimentin in MSCs. In the animal experiment, MSCs could favor VX2 bladder tumor growth and up-regulate the expression of TGFβ1, EGF, FAPa, MMP9 in VX2 tumor tissue. However, when TGFβ1 receptor or Smad2 of MSCs was blocked, the above effects were attenuated.

**Conclusions:** Under the induction of tumor microenvironment, MSCs can differentiate into myofibroblasts and then affect tumor interstitial microenvironment remodeling. This process is mediated by TGFβ1-Smad2 pathway.

## Introduction

Mesenchymal stem cells (MSCs) are widely distributed in the body, mainly in the bone marrow. They have been widely used in tissue engineering, cells and gene therapy. Regarding to how the target organ induces MSCs' migration, it is generally thought that the extracellular signals emitted by the injured organ regulate this process, and various chemokine receptors expressed by MSCs are closely related to the migration of MSCs^1^, ^2^. After the migration of MSCs to target organs, their further differentiation is environmental-dependent^3^,^4^.

As a rapidly growing and metabolically active disease, tumor can also induce MSCs to differentiate into cells that are conducive to tumor growth and invasion. Current research results have indeed confirmed this hypothesis^5^. Studeny et al transferred adenovirus vectors carrying the β-interferon gene into MSCs and found a large number of MSCs in tumor tissue^6^. These experimental results show that tumor cells do have the ability to induce the migration of MSCs. Other related studies also found similar results^7^, ^8^. Furthermore, studies also found that MSCs could promote tumor growth and metastasis^9^-^11^.

Our previous research results showed that under the induction of tumor microenvironment, MSCs could differentiate into myofibroblasts, and further accelerate the development of tumors by promoting tumor interstitial microenvironment remodeling^10^, ^12^. The mechanism of tumor-induced differentiation of MSCs into myofibroblasts remains unclear. In the study of lysophosphatidic acid-induced differentiation of MSCs into myofibroblasts, Jeon et al found that blocking the expression of Smad2/3 would significantly reduce the expression of α-SMA, while blocking the binding of transforming growth factorβ1(TGFβ1) to its receptor could reduce the α- SMA expression and Smad2 phosphorylation^13^. This result suggests that TGFβ1-Smad signaling pathway plays an important role in the differentiation of MSCs into myofibroblasts. In view of the important role of myofibroblasts in the remodeling of tumor interstitial microenvironment, the blockage of TGFβ1-Smad signaling pathway in MSCs may simultaneously affect the remodeling of tumor interstitial microenvironment and further affect the development of tumor. This study mainly validated the above hypothesis. And the remodeling of tumor interstitial microenvironment was shown by the expression of TGFβ1, epidermal growth factor (EGF), fibroblast activation protein alpha (FAPa), matrix metalloprotein 9 (MMP9).

## Materials and Methods

### The design of the study

MSCs have been proved to be able to differentiate into myofibroblasts and then affect tumor stroma remodeling, but the mechanism is not clear. This study was aimed to ascertain whether TGFβ1-Smad pathway was involved in this process. In order to verify the hypothesis, we designed an* in vitro* experiment and an animal experiment. The* in vitro* experiment aimed to find out whether TGFβ1-Smad pathway was involved in the VX2 culture supernatant-induced differentiation of MSCs to myofibroblasts. The animal experiment aimed to discover whether TGFβ1-Smad pathway was involved in the process that MSCs could affect tumor stroma remodeling.

### Animals

Fifty male New Zealand white rabbits (3 months old, weight 1.5-2.0kg) were purchased from Shandong Academy of Agricultural Sciences. The animal protocols were approved by the Institutional Animal Care and Use Committee of Qilu Hospital, Shandong University.

### The groups of *in vitro* experiment

There were five groups for the* in vitro* experiment, named as control A, control B, control C, test A, test B, respectively. For group control A, F2 passage MSCs were cultured in DMEM-LG with 10% calf-serum, 30% VX2 culture supernatant. For group control B, F2 passage MSCs, which had been transfected by blank liposomes, were cultured in DMEM-LG with 10% calf-serum, 30% VX2 culture supernatant. For group control C, F2 passage MSCs were cultured in DMEM-LG with 10% calf-serum. For group test A, F2 passage MSCs, which had been transfected with siRNA targeting TGFβ1 receptor by liposomes, were cultured in DMEM-LG with 10% calf-serum, 30% VX2 culture supernatant. For group test B, F2 passage MSCs, which had been transfected with siRNA targeting Smad2 by liposomes, were cultured in DMEM-LG with 10% calf-serum, 30% VX2 culture supernatant. The expression of α-SMA and Vimentin in MSCs was detected by westernblot 14 days later. 10 samples of each group, 10^7^ MSCs in each sample, were tested.

### The groups of animal experiment

Fifty male New Zealand white rabbits were randomly divided into control 1, control 2, control 3, test 1, test 2, with ten rabbits in each group. For group control 1, the cell suspension, containing 10^6^ autologous MSCs and 10^6^ VX2, was injected into the bladder submucosa. For group control 2, the cell suspension, containing 10^6^ autologous MSCs transfected by blank liposomes and 10^6^ VX2, was injected into the bladder submucosa. For group control 3, the cell suspension, containing 10^6^ VX2, was injected into the bladder submucosa. For group test 1, the cell suspension, containing 10^6^ autologous MSCs transfected with siRNA targeting TGFβ1receptor by liposomes and 10^6^ VX2, was injected into the bladder submucosa. For group test 2, the cell suspension, containing 10^6^ autologous MSCs transfected with siRNA targeting Smad2 by liposomes and 10^6^ VX2, was injected into the bladder submucosa.

The animals were sacrificed 4 weeks later. The expression of TGFβ1, EGF, FAPa, MMP9 were detected by westernblot, and the tumor size was recorded.

### MSCs isolation, cultivation and identification

The process of MSCs isolation, cultivation and identification was the same as we previously described^12^. MSCs with CD34(-), CD44(+) and CD45(-) were used for study.

### VX2 tumor cells isolation and cultivation

The VX2 tumor carrier rabbit was presented by Radiology Department of Qilu Hospital. The process of VX2 tumor cells isolation and cultivation was the same as we previously described 12.

### Tumor inoculation procedure

**Figure 1:**
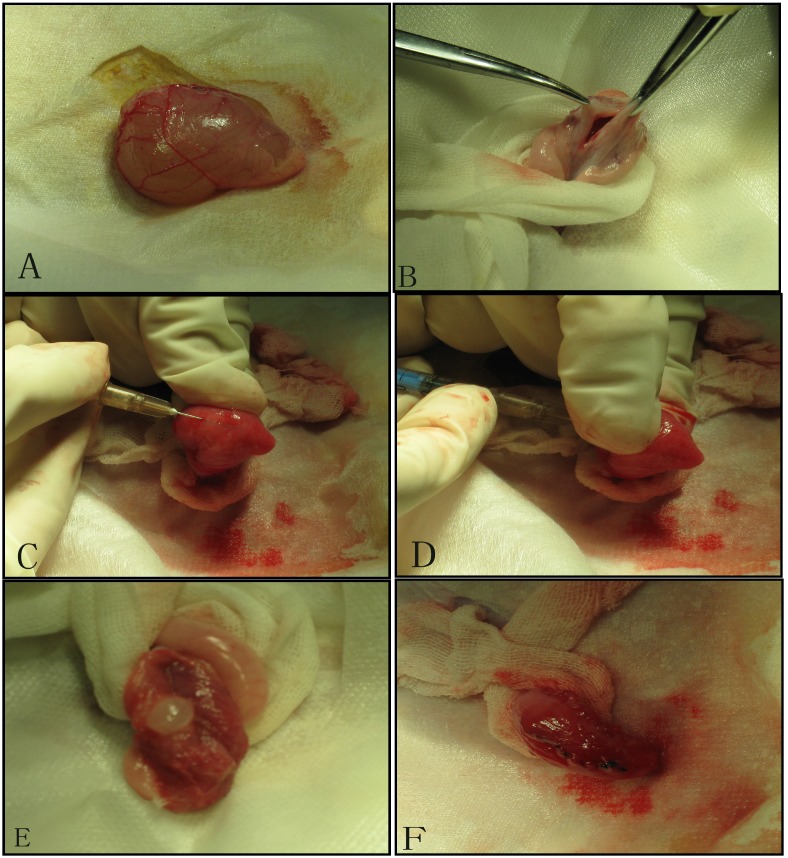
Tumor inoculation procedure: A. Exposure the bladder; B. Cystotomy along the midline; C. 1 ml needle (containing 300 ul of cell suspension) punctures into the mucosa on the side wall of the bladder; D. After about 1.5cm of immersion in the submucosa of the bladder, the thumb of the left hand presses against point of the needle, and 300ul of cell suspension is injected under the mucosa; E. Blisters formed by cell suspension after injection; F. Close the bladder with absorbable suture.

We established tumor model by injecting mixed cell suspensions under the bladder mucosa. After the exposure of the urinary bladder through the lower abdominal incision under sterile conditions, we made a small incision (0.8cm) in the bladder wall, and perpendicularly inoculated the cell suspension into the bladder wall. Then bladder wall and abdominal wall were stitched in turn^12^.(Figure 1)

### Cell transfection

According to LipofectamineTM 2000 liposome transfection reagent instruction (Lipofect2000, Invitrogen), F2 passage MSCs were digested by pancreatic enzyme, and then were counted. They were inoculated at 2x10^5^/mL. When fusion of cell growth was 70%-80%, OD SiRNA diluted by OptiMEM® and LipofectamineTM 2000 diluted by OptiMEM® Medium were mixed, and incubated for 20 min; Mixture was added to each hole, and incubated at 37 ℃ and 5% CO2, saturated humidity. After 9 hr they were cultured by DMEM containing 10% FBS/F-12 media, and then were screened and cultured.

### Western blotting

Briefly, the protein concentrations of all samples were measured by BCA protein assay kit (Pierce, USA) after protein extraction. After boiled for 5 min, the protein samples were fractionated by SDS-PAGE (10-15% polyacrylamide gels) and transferred to PVDF membrane (Millipore, Bedford, MA, USA). The samples were blocked with milk powder for 1 hr at room temperature and then incubated with primary antibodies (Abcam China) as well as calcineurin and NFATc3 (Santa Cruz Biotechnology Inc., Santa Cruz, CA USA) at 4°C overnight. After washing, the membranes were incubated with a secondary antibody (Santa Cruz Biotechnology Inc., Santa Cruz, CA USA) for 1 hr at room temperature. Western blot bands were quantified using Gel analysis system by measuring the integrated optical density (IOD). The protein expression intensity was quantified by relative optical density (ROD). The ROD was defined as protein IOD/actin IOD.

### Statistical analysis

All values were expressed as mean ± standard deviation (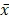
±S). SPSS 17.0 was used to deal with the data, and t test was used to determine statistical differences between two groups. P<0.05 was considered as significant difference.

## Results

### The *in vitro* experiment showed that blocking the expression of TGFβ1 receptor or Smad2 affected the differentiation of MSCs to myofibroblast

**Figure 2:**
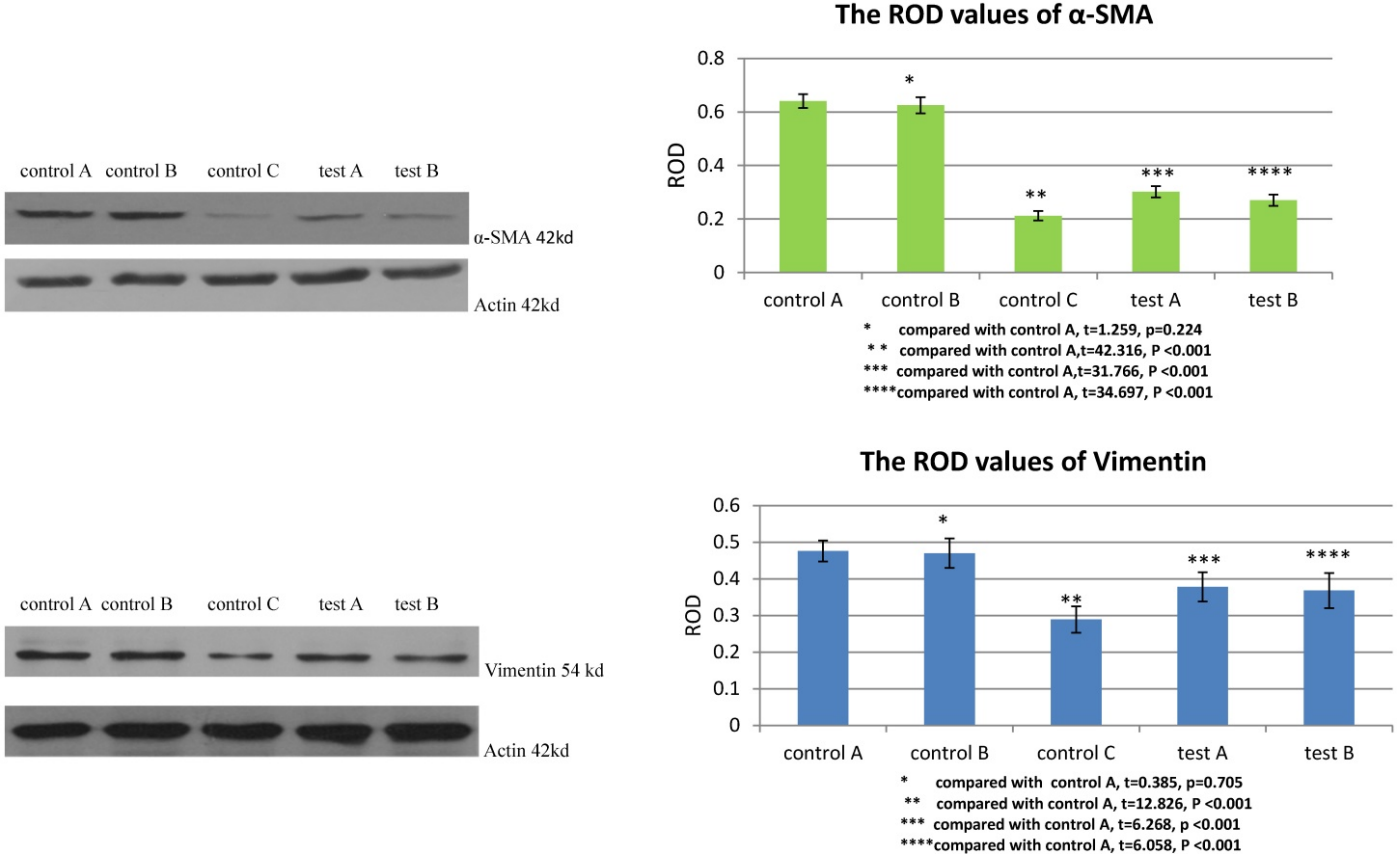
The expression of α-SMA and Vimentin in MSCs was detected by westernblot. Control B was used to test whether or not liposome could affect the result. Compared with control A, p>0.05 indicated liposome did not affect the result. The comparison between group control C and group control A indicated that VX2 culture supernatant could induce the expression of α-SMA and Vimentin. Blockage of the expression of TGFβ1 receptor (group test A) or Smad2 (group test B) would both lead to decreased expression of α-SMA and Vimentin.

The ROD values of α-SMA and Vimentin were 0.212±0.018 and 0.289±0.036 when MSCs were cultured with DMEM-LG in group control C. When MSCs were cultured with DMEM-LG and 30% VX2 culture supernatant in group control A, the ROD values of α-SMA and Vimentin were both significantly increased (0.641±0.026, 0.476±0.029), which indicated VX2 culture supernatant could induce the differentiation of MSCs to myofibroblasts. However, compared with group control A, the ROD values of α-SMA and Vimentin were both significantly decreased when the expression of TGFβ1 receptor was blocked by siRNA in group test A (0.302±0.021, 0.378±0.040). Besides, both the ROD values of α-SMA and Vimentin were significantly decreased when the expression of Smad2 was blocked by siRNA in group test B (0.270±0.021, 0.368±0.048). The results indicated that both TGFβ1 receptor and Smad2 were involved in the differentiation of MSCs to myofibroblasts.(Figure 2)

### TGFβ1 receptor and Smad2 participate in the MSCs-induced acceleration of VX2 tumor growth

**Figure 3:**
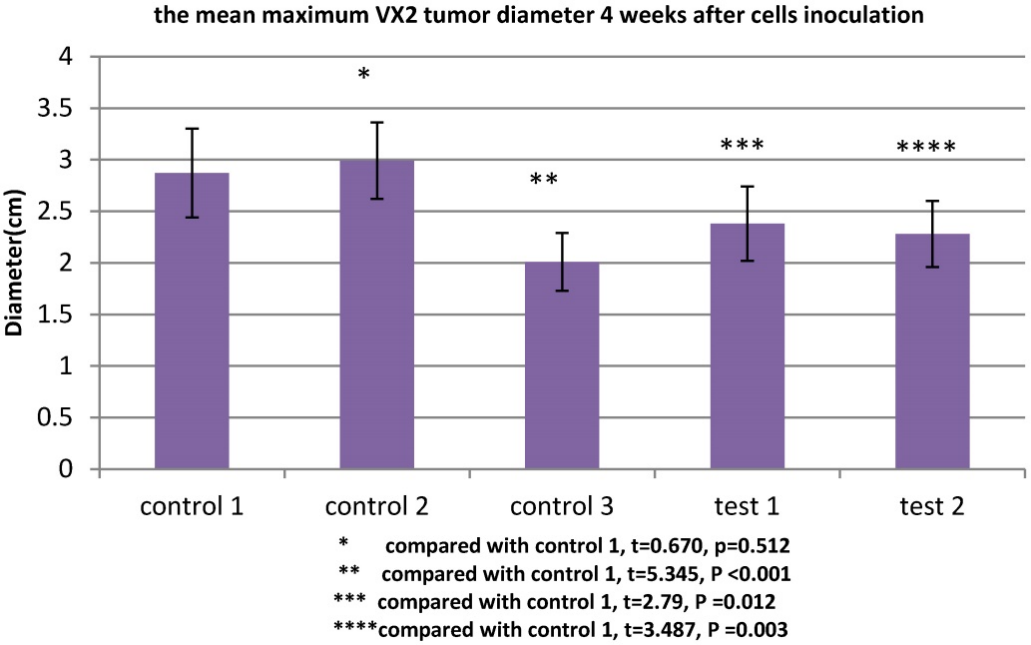
The maximum diameter of VX2 bladder tumor 4 weeks after tumor inoculation. Control 2 was used to test whether or not liposome could affect the result. Compared with control 1, p>0.05 indicated liposome did not affect the result. The comparison between group control 3 and group control 1 indicated that MSCs were conducive to VX2 tumor growth. The expression of TGFβ1 receptor and Smad2 of MSCs were blocked in group test 1 and group test 2, respectively. Compared with group control 1, the mean maximum tumor diameter was significantly decreased in group test 1 and group test 2

All rabbits were sacrificed 4 weeks after tumor inoculation. The maximum diameter was recorded. The mean maximum diameter of group control 3 was 2.01±0.28cm. When VX2 cells and MSCs were co-inoculated in group control 1, the mean maximum diameter was significantly increased (2.87±0.43cm), which indicated that MSCs were conducive to VX2 tumor growth. In group test 1, the expression of TGFβ1 receptor of MSCs was blocked, then these cells were co-inoculated with VX2 cells. Compared with group control 1, the mean maximum diameter was significantly decreased (2.38±0.36cm). The same tendency was also found in group test 2. After the expression of Smad2 of MSCs was blocked, these MSCs were co-inoculated with VX2 cells. Compared with group control 1, the mean maximum diameter was also significantly decreased (2.28±0.32cm). (Figure 3)

### TGFβ1 receptor and Smad2 mediated the facilitation of MSCs to the expression of TGFβ1 and EGF in VX2 tumor tissue

**Figure 4:**
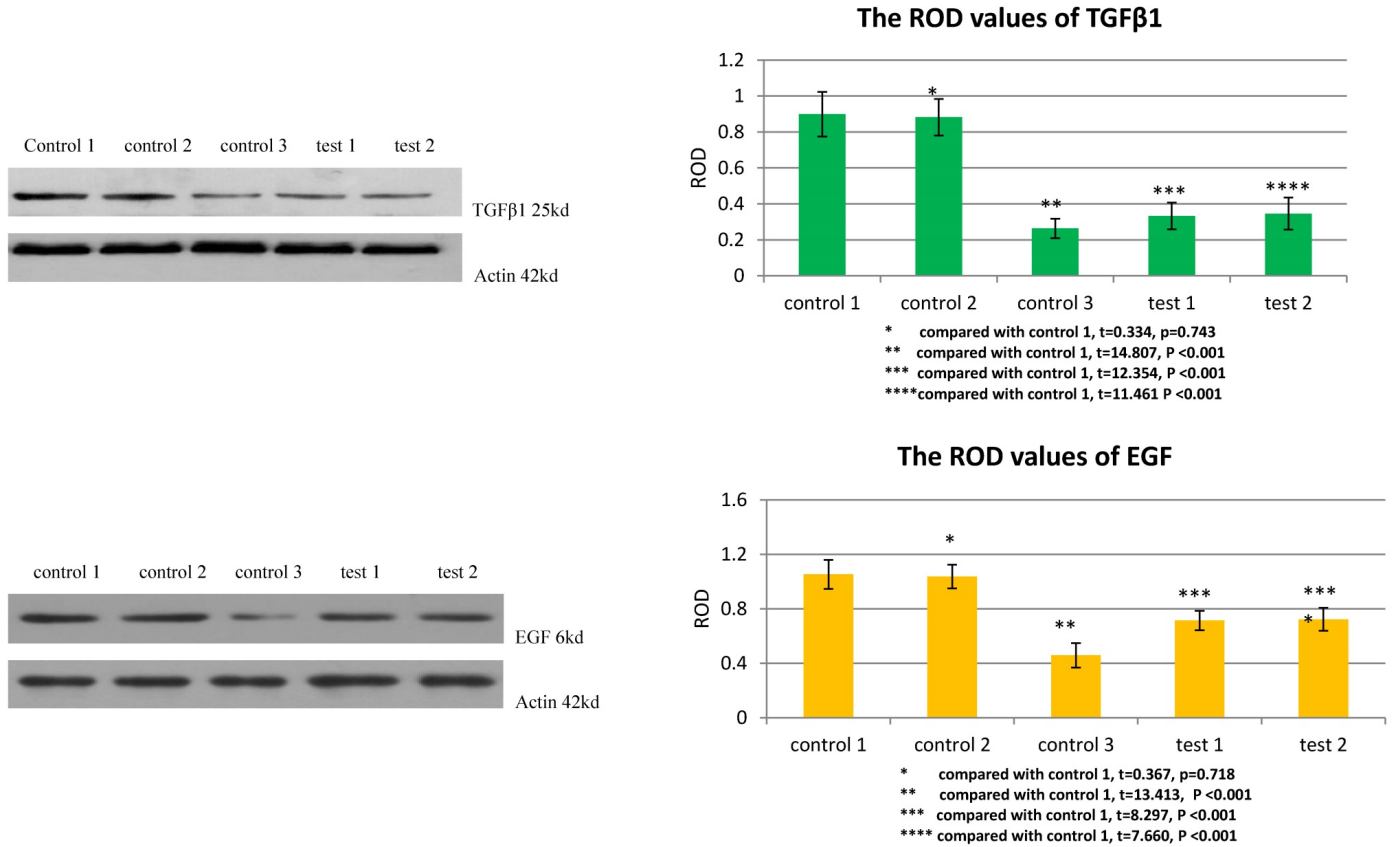
The expression of TGFβ1 and EGF in vx2 tumor tissue was detected by westernblot. Control 2 was used to test whether or not liposome could affect the result. Compared with control 1, p>0.05 indicated liposome did not affect the result. The comparison between group control 3 and group control 1 indicated that MSCs could enhance the expression of TGFβ1 and EGF. Blockage of the expression of TGFβ1 receptor (group test 1) or Smad2 (group test 2) would both lead to decreased expression of TGFβ1 and EGF.

The ROD values of TGFβ1 and EGF were 0.264±0.054 and 0.459±0.090 in group control 3. When VX2 cells and MSCs were co-inoculated in group control 1, the ROD values of TGFβ1 and EGF were both significantly increased (0.899±0.124, 1.053±0.107), which indicated that MSCs facilitated the expression of these two growth factors in VX2 tumor tissue. Compared with group control 1, the ROD values of TGFβ1 and EGF were both significantly decreased (0.333±0.074, 0.714±0.072) when the expression of TGFβ1 receptor of MSCs was blocked in group test 1. Moreover, both the ROD values of TGFβ1 and EGF were significantly decreased when the expression of Smad2 of MSCs was blocked in group test 2 (0.346±0.089, 0.723±0.084). The results indicated that both TGFβ1 receptor and Smad2 were involved in the process that MSCs facilitated the expression of TGFβ1 and EGF in VX2 tumor tissue. (Figure 4)

### TGFβ1 receptor and Smad2 mediated the facilitation of MSCs to the expression of FAPa and MMP9 in VX2 tumor tissue

**Figure 5:**
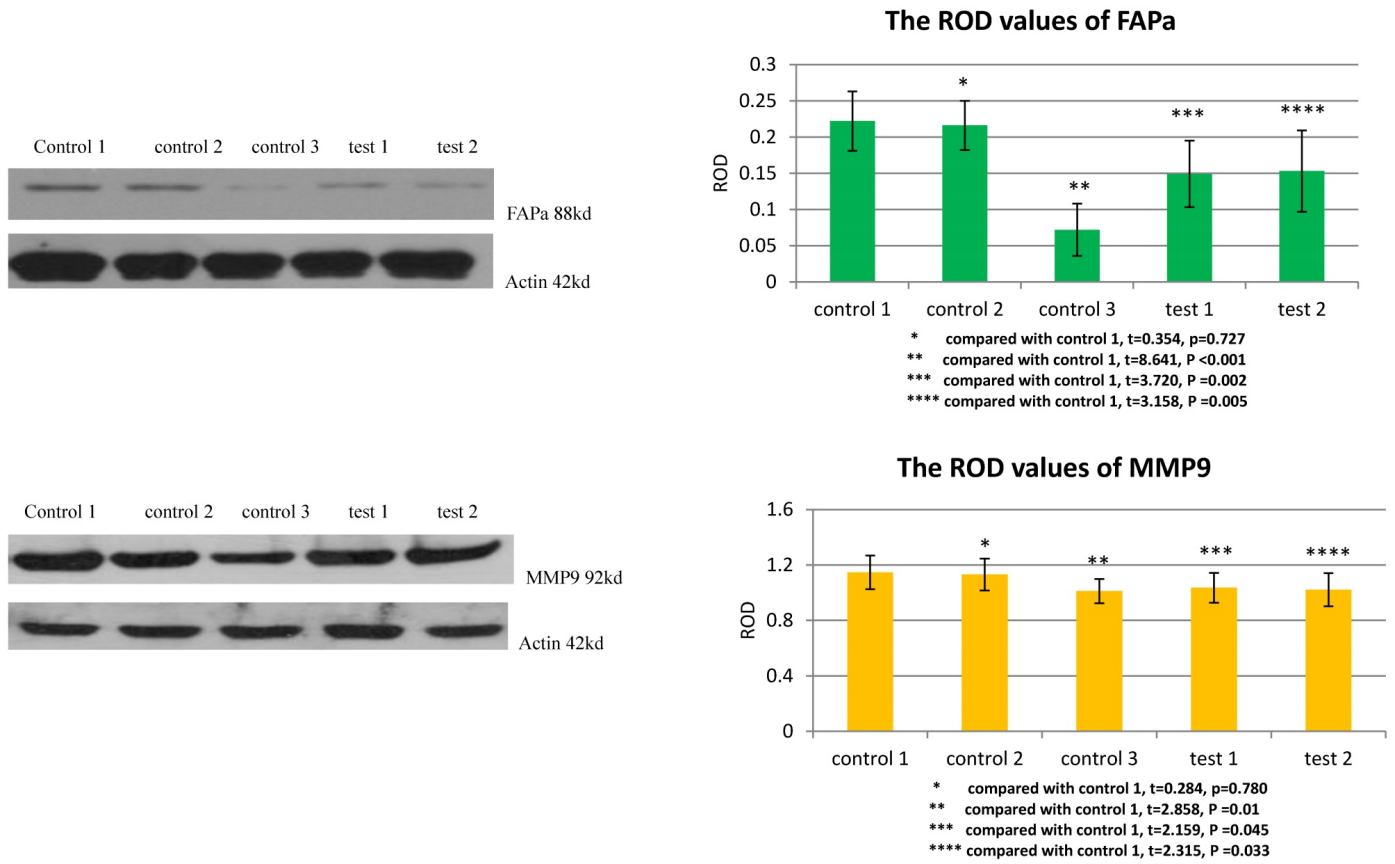
The expression of FAPa and MMP9 in vx2 tumor tissue was detected by westernblot. Control 2 was used to test whether or not liposome could affect the result. Compared with control 1, p>0.05 indicated liposome did not affect the result. The comparison between group control 3 and group control 1 indicated that MSCs could enhance the expression of FAPa and MMP9. Blockage of the expression of TGFβ1 receptor (group test 1) or Smad2 (group test 2) would both lead to decreased expression of FAPa and MMP9.

The ROD values of FAPa and MMP9 were 0.072±0.036 and 1.012±0.087 in group control 3. When VX2 cells and MSCs were co-inoculated in group control 1, the ROD values of FAPa and MMP9 were both significantly increased (0.222±0.041, 1.147±0.121). When the expression of TGFβ1 receptor of MSCs was blocked in group test 1, the ROD values of FAPa and MMP9 were both significantly decreased (0.149±0.046, 1.036±0.108) compared with group control 1. And both The ROD values of FAPa and MMP9 were significantly decreased when the expression of Smad2 of MSCs was blocked in group test 2 (0.153±0.056, 1.022±0.120). The results indicated that both TGFβ1 receptor and Smad2 were involved in the process that MSCs facilitated the expression of FAPa and MMP9 in VX2 tumor tissue. (Figure 5)

## Discussion

Activation of microenvironment is a critical step for tumor growth and development^14^-^16^. Activated mesenchymal cells produce a large amount of extracellular matrix components, growth factors, and matrix remodeling proteins, thereby forming a microenvironment that is conducive to tumor growth and proliferation. These mesenchymal cells mainly include fibroblasts, myofibroblasts, endothelial cells, and some immune cells^17^. Although all of the above cells play a role in the growth and development of tumors, it is currently believed that myofibroblasts are especially important for tumor growth, invasion and metastasis^18^-^20^. In the activated interstitial microenvironment, myofibroblasts produce some proteases such as fibroblast activation protein, metalloproteinases, urokinase, plasminogen activator. In addition, they also synthesize some extracellular matrix components such as collagen I, collagen III, fibronectin, mucin, polysaccharide proteins. Some reports suggest that myofibroblasts can secrete certain growth factors to promote the development of cancer, such as TGFβ1, EGF, platelet-derived growth factor, fibroblast growth factor, hepatocyte growth factor, keratinocyte growth factor, stem cell factor^21^, ^22^.

In the tumor stroma, myofibroblasts are mainly derived from fibroblasts. Since many scholars believe that the fibroblasts in the granulation tissue can be derived from some progenitor cells in the circulation^23^-^25^, Alexis et al therefore believe that the circulating cells will also migrate to tumor tissue and differentiate into myofibroblasts when the tumor grows to a certain size and local fibroblasts are not sufficient^26^. Ishii's findings further indicate that myofibroblasts in tumor stroma are derived from bone marrow, and that the higher the tumor stage, the greater the number of myofibroblasts derived from the bone marrow^27^. However, this study only showed that myofibroblasts in tumor stroma could also derive from the bone marrow but did not specify which cells they derived from. Jeon's findings indicate that lysophosphatidic acid secreted by tumors can induce the differentiation of MSCs into myofibroblast-like cells^13^. Mishra found that the expression of α-SMA, a marker of myofibroblast, was significantly increased in bone marrow mesenchymal cells induced by tumor-conditioned medium, and the induced MSCs significantly promoted tumor growth both* in vitro* and *in vivo*^28^. Therefore, at least part of myofibroblasts in tumor tissue should derive from bone marrow MSCs. In addition to bone marrow, MSCs are widely distributed in other parts of the body. Therefore, myofibroblasts in tumors may also derive from MSCs in other sites. In addition, Bagley's results indicate that MSCs are mainly distributed in tumor stroma after entering tumor tissue, and they mainly differentiate into myofibroblast precursor cells, fibroblasts^29^. In view of the important role of myofibroblasts in tumor growth, invasion, and metastasis, one of the mechanisms by which MSCs facilitate tumor growth is to promote tumor growth by differentiated myofibroblasts.

Smad2/Smad3 is the first signaling molecule in TGFβ1- Smad signaling pathway. As a direct substrate of the TGFβ superfamily, Smad2/Smad3 plays a key mediating role in the transmission of TGFβ1 signaling from cytoplasm to nucleus^30^, ^31^. In the previous studies, we found that MSCs could differentiate into myofibroblasts under the induction of tumor microenvironment, and further promote the growth and development of tumors by promoting remodeling of tumor interstitial microenvironment^10^, ^12^. In this study, we also found that MSCs could differentiate into myofibroblasts under the induction of tumor cell culture supernatant. Blocking the expression of TGFβ1 receptor or Smad2 in MSCs can significantly down-regulate the expression of α-SMA and Vimentin. This indicates that TGFβ1-Smad signaling pathway is involved in the differentiation of MSCs into myofibroblasts. We further investigated whether TGFβ1-Smad signaling pathway was involved in the process that MSCs facilitated tumor growth and tumor interstitial microenvironment remodeling. We found that blocking the expression of TGFβ1 receptor or Smad2 in MSCs could down-regulate the tumor growth promoting effect of MSCs, and significantly down-regulate the expression of TGFβ1, EGF, FAPa and MMP9, which were closely related to the activation and remodeling of tumor interstitial microenvironment. We speculate that one of the reasons for this result is that blocking TGFβ1-Smad signaling pathway affects the differentiation of MSCs into myofibroblasts.

Although most studies indicate that MSCs facilitate tumor growth and metastasis, some studies indicate that MSCs can inhibit tumor development. However, the vast majority of these studies are the results of* in vitro* cell experiments or immunodeficient mice, and the MSCs used are allogeneic. So one merit of this study is that the animals used in this study are normal rabbits and MSCs used in every rabbit are autologous, which avoids the immune interference on the study results.

## Conclusions

Under the induction of tumor microenvironment, MSCs can differentiate into myofibroblasts and then affect tumor interstitial microenvironment remodeling. This process is mediated by TGFβ1-Smad2 pathway.

## Abbreviations

MSCs: Mesenchymal stem cells
TGFβ1: transforming growth factor β1
EGF: epidermal growth factor
FAPa: fibroblast activation protein alpha
MMP9: matrix metalloprotein 9
PBS: phosphate buffered saline
DMEM-LG: dulbecco's modified eagle medium-low glucose
IOD: intergrated optical density
ROD: relative optical density
